# Mapping the MOB proteins’ proximity network reveals a unique interaction between human MOB3C and the RNase P complex

**DOI:** 10.1016/j.jbc.2023.105123

**Published:** 2023-08-01

**Authors:** Islam E. Elkholi, Jonathan Boulais, Marie-Pier Thibault, Hong-Duc Phan, Amélie Robert, Lien B. Lai, Denis Faubert, Matthew J. Smith, Venkat Gopalan, Jean-Franҫois Côté

**Affiliations:** 1 Montreal Clinical Research Institute (IRCM), Montreal, Quebec, Canada; 2 Molecular Biology Programs, Université de Montréal, Montreal, Quebec, Canada; 3 Department of Anatomy and Cell Biology, McGill University, Montreal, Quebec, Canada; 4 Department of Chemistry & Biochemistry, Center for RNA Biology, The Ohio State University, Columbus, Ohio, USA; 5 Institute for Research in Immunology and Cancer, Université de Montréal, Montreal, Quebec, Canada; 6 Department of Biochemistry and Molecular Medicine, Université de Montréal, Montreal, Quebec, Canada

**Keywords:** MOB proteins, MOB2, MOB4, MOB3C, proteomics, BioID, proximity labeling techniques, RNase P complex

## Abstract

Distinct functions mediated by members of the monopolar spindle-one-binder (MOB) family of proteins remain elusive beyond the evolutionarily conserved and well-established roles of MOB1 (MOB1A/B) in regulating tissue homeostasis within the Hippo pathway. Since MOB proteins are adaptors, understanding how they engage in protein–protein interactions and help assemble complexes is essential to define the full scope of their biological functions. To address this, we undertook a proximity-dependent biotin identification approach to define the interactomes of all seven human MOB proteins in HeLa and human embryonic kidney 293 cell lines. We uncovered >200 interactions, of which at least 70% are unreported on BioGrid. The generated dataset reliably recalled the *bona fide* interactors of the well-studied MOBs. We further defined the common and differential interactome between different MOBs on a subfamily and an individual level. We discovered a unique association between MOB3C and 7 of 10 protein subunits of the RNase P complex, an endonuclease that catalyzes tRNA 5′ maturation. As a proof of principle for the robustness of the generated dataset, we validated the specific interaction of MOB3C with catalytically active RNase P by using affinity purification–mass spectrometry and pre-tRNA cleavage assays of MOB3C pulldowns. In summary, our data provide novel insights into the biology of MOB proteins and reveal the first interactors of MOB3C, components of the RNase P complex, and hence an exciting nexus with RNA biology.

The highly conserved mammalian monopolar spindle-one-binder (MOB) family of proteins is comprised of seven members and is subdivided into four subfamilies: MOB1A/B, MOB2, MOB3A/B/C, and MOB4. This family regulates cell cycle/division dynamics, DNA repair, tissue growth, and morphogenesis, in addition to cytoskeletal organization (1, 2, 3). These notions globally elected this family as potential players in growth-related disorders such as cancer (4, 5, 6, 7, 8). However, these functions are revealed only in the context of the best characterized members, namely MOB1A/B as *bona fide* regulators of the Hippo pathway, followed by the relatively explored members, MOB2 as a regulator of nuclear dbf2-related kinase activity, and MOB4 as a component of the striatin-interacting phosphatase and kinase (STRIPAK) complex (9, 10, 11). The MOB3 subfamily is poorly characterized in terms of binding partners and function beyond speculations based on unvalidated screens’ predictions (1). This gap in knowledge might be partially attributed to (i) the lack of mammalian diversity (the absence of orthologs for this subfamily members) in the model organisms where the MOBs were initially discovered and characterized phenotypically such as *Saccharomyces cerevisiae* (12, 13) (has two MOB proteins: Mob1p and Mob2p) and *Drosophila melanogaster* (14) (has four MOB proteins: dMob1, dMob2, dMob3, dMob4) or (ii) considering the three MOB3 proteins as a single entity given the structural similarity (15).

MOBs are small ∼20 kDa single-domain proteins that overall share 17 to 96% structural similarity between different members and are thought of as scaffolds or adaptor proteins that mediate their functions mainly through engaging with and assembling protein complexes (1, 3). Therefore, different proteomic approaches aiming at revealing protein–protein interactions (PPIs) have been leveraged to reveal the MOB proteins’ interactomes but mainly within the borders of the Hippo and STRIPAK complexes (11, 16, 17). Despite the advances from these previous studies, including insights into the crosstalk between MOB1 and MOB4 within the Hippo pathway and STRIPAK complex (17, 18), a systematic comparison of the interactomes of all seven MOBs in the same cellular context has not been undertaken before. The work described here addresses this gap.

Here, we harnessed the biotinylation-dependent proximity labeling (biotinylation identification [BioID]) approach (19, 20, 21), which bypasses different limitations of standard PPI-profiling techniques (22), to explore the global interactome of the MOB proteins in two different cell lines with an intent to focus on the less characterized MOB3s. We reveal the common and unique interactors among the MOB subfamilies and between subfamily members. Unexpectedly, we discovered that MOB3C exists in the vicinity of 7 of 10 protein subunits of the RNase P complex, an endoribonuclease that catalyzes the cleavage of 5′ leader from precursor tRNAs (23, 24). Further investigations confirmed that MOB3C, but not MOB1A, interacts with a catalytically active RNase P complex. Our results provide new insights into the MOB proteins’ interactors and hence shedding light on their functional diversity beyond the view of being kinase activators. Importantly, we uncover a novel potential connection between MOB3C and RNA biology.

## Results

### BioID proximity labeling screens for mapping the global interactome of MOB proteins

**Figure 1:**
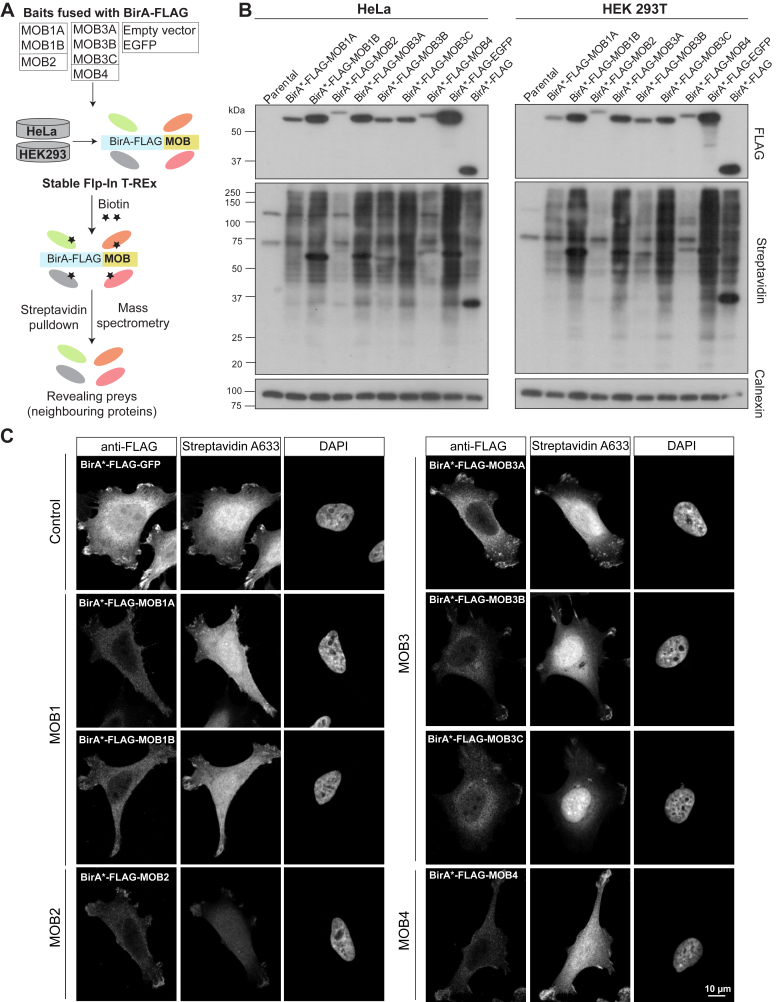
**BioID proximity labeling screens for the MOB proteins.***A*, schematic outline of the BioID screens’ pipeline. *B*, Western blot analysis of the parental and BioID bait-expressing Flp-In T-REx HeLa and HEK 293T cells. *C*, confocal microscopy images of the bait’s expression (anti-FLAG) and biotinylation (streptavidin) patterns for the seven MOB proteins and a control condition (cells expressing BirA-FLAG-EGFP) in HeLa cells. *B* and *C*, cells were treated with tetracycline (to induce expression) and biotin (to induce biotinylation) for 24 h. BioID, biotinylation identification; EGFP, enhanced GFP; HEK, human embryonic kidney cell line; MOB, monopolar spindle-one-binder.

We exploited a BioID pipeline to reveal the MOB proteins’ potential interactors on a global scale to assess how they are functionally related as this remains elusive (2, 3). BioID depends on fusing a bait, here the seven MOBs, to an abortive mutant form of the biotin ligase BirA (BirA∗) that stimulates the biotinylation of proteins within approximately 10 nm vicinity of the bait (19). As we and others previously demonstrated (19, 20, 21, 25), this unbiased approach can (i) capture transient interactors in addition to those that may be missed because of solubility-related caveats (*i.e.*, insoluble cellular structures and/or use of harsh lysis conditions for disrupting interactions at the cell membrane for instance (22)) and (ii) map the spatial landscape of the bait-specific signaling pathways. To this end, we generated tetracycline-inducible human embryonic kidney 293 (HEK293) and HeLa Flp-In T-REx cells expressing either BirA∗-FLAG-MOB (for the seven human MOB proteins; Table S1), BirA∗-FLAG, or BirA∗-FLAG-enhanced GFP (EGFP), where the latter two serve as negative controls (Fig. 1*A*). We chose an N-terminal tagging strategy to generate the BirA∗-FLAG–MOB fusion proteins given this strategy’s previous success for MOB1A/B (16). The expression of the different baits (MOB proteins or controls) in HEK293 and HeLa cells as well as the overall cellular biotinylation profiles was validated by Western blotting (Fig. 1*B*) and immunofluorescence (Fig. 1*C*).

**Figure 2:**
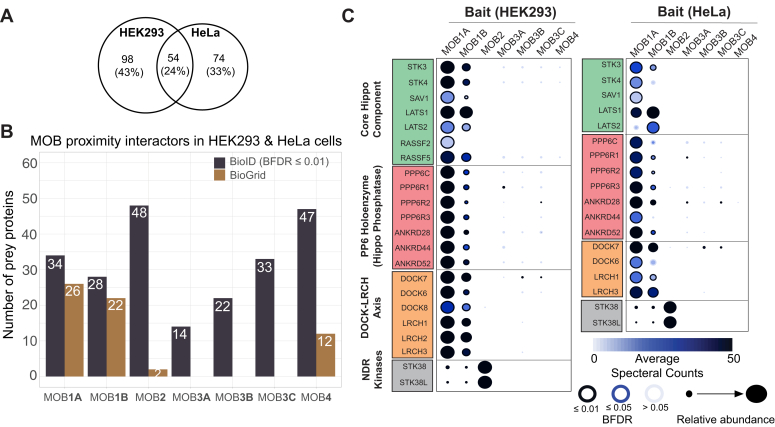
**BioID recalls the *bona fide* MOB protein interactors and extends their proximity network.***A*, Venn diagrams showing the number of MOB preys identified in the two cell lines used in BioID. *B*, histogram demonstrating an enumeration of interactors identified for every MOB protein in our BioID screens (BFDR ≤ 0.01) in addition to a representation of the BioGrid recalls. *C*, dot plots highlighting the *bona fide* interactors of MOB1A/B and MOB2 in HeLa and HEK293 cells. BFDR, Bayesian false discovery rate; BioID, biotinylation identification; HEK293, human embryonic kidney 293 cell line; MOB, monopolar spindle-one-binder.

The BioID screens revealed 226 proximity interactors for the MOB family, 54 of which were shared between HeLa and HEK293 cells (Fig. 2*A* and Table S2). Such overlap between the two cell lines (54 of 226 interactors; 24% of the dataset size) is in line with previously reported datasets for different proteins (20). Among the revealed interactions, 62 (27%) were previously reported in BioGrid (Fig. 2*B*). These previously mapped interactions were mainly for MOB1A/B (48 interactions) and MOB4 (12 interactions) with none for MOB3A/B/C. MOB1s are an integral part of the Hippo pathway where the kinase MST1/2 phosphorylates MOB1 that binds to the kinase LATS (large tumor suppressor) allowing its autophosphorylation on the activation segment. MST1/2 also phosphorylate the hydrophobic motif of LATS for full activation. LATS phosphorylates and inhibits the activity of YAP1 (Yes-associated protein 1), the Hippo pathway effector (26). Indeed, we found that in both HEK293 and HeLa cells, all the pathway’s core components (LATS1/2 and STK3/4) and phosphatase (PP6 holoenzyme) were retrieved (16) (Fig. 2*C*). Furthermore, the previously reported cytoskeleton-associated DOCK6–8 and LRCH1–3 proteins were also recalled (16). Similarly, the MOB2 *bona fide* interactor nuclear dbf2-related kinases, STK38 and STK38L, were captured among top hits for MOB2 in our datasets (3, 27) (Fig. 2*C*). These findings gave confidence that our screens could reliably identify new protein interaction partners of MOB3 proteins, our primary focus.

### Global overview of the MOBs’ interactomes

**Figure 3:**
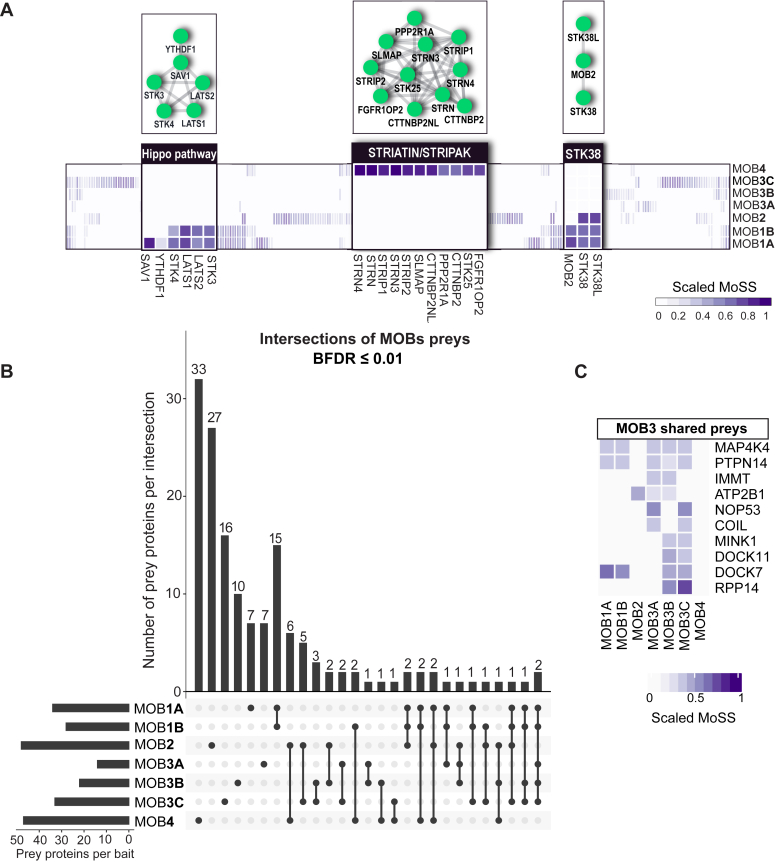
**Overview of the interactome of the MOB subfamilies.***A*, heatmap highlighting the relative MoSS score for the *bona fide* interactors of MOB1A/B, MOB2, and MOB4. *B*, an UpSet plot enumerating intersections of nonredundant proximal interactions identified for different MOB baits in either HeLa or HEK293 cells by BioID. *C*, heatmap highlighting the preys shared between MOB3A/B/C and other MOB subfamilies in addition to their individual common preys. HEK293, human embryonic kidney 293 cell line; MOB, monopolar spindle-one-binder; MoSS, MOB specificity score.

Toward a holistic and individual MOB protein-based analysis, we applied a method that assigns a specificity score (MOB specificity score [MoSS]; see Experimental procedures section) for each prey toward a MOB bait after combining the datasets of the two cell lines together. This approach reassuringly pinpointed the Hippo pathway components and striatin (STRIPAK complex) as top specific interactors for MOB1A/B and MOB4, respectively, as outlined previously and established in the previous literature (11, 16) (Fig. 3*A*). Exploiting the MoSS metric, we generated an UpSet plot to globally compare the interactome of all the MOBs combined (Fig. 3*B*). The highest number of shared proteins between MOBs was 15 proximal proteins between MOB1A and MOB1B. We then turned our focus to the MOB3 subfamily and first assessed whether they share neighboring proteins with other MOB subfamilies. The MOB3 subfamily shared only two proximal proteins (MAP4K4 and PTPN14) with the MOB1A/B subfamily (Fig. 3*C*). Interestingly, both proteins are noncanonical Hippo pathway regulators, beyond the core components outlined previously. A kinome-focused screen for MST1/2-independent kinases of LATS1/2 revealed six candidate kinases, including MAP4K4 that was further demonstrated to phosphorylate the hydrophobic motif of LATS and consequently inactivate YAP (28). Meanwhile, PTPN14, a nonreceptor protein tyrosine phosphatase, associates with and inhibits YAP activity (16, 29, 30, 31).

We then assessed whether MOB3A, MOB3B, and MOB3C share additional neighboring proteins. MOB3A shared IMMT, a subunit of the mitochondrial contact site and cristae organizing system complex (32), with MOB3B. MOB3A and MOB3B further shared the calcium transporter/pump ATP2B1 (33) with MOB2. However, these two shared candidate interactors were captured at low abundance (Fig. 3*C* and Table S2). MOB3A further uniquely shared two nuclear proteins, NOP53 and COIL, with MOB3C (Fig. 3*C*). NOP53 is involved in ribosomal biogenesis and regulating the P53 pathway (34, 35), whereas COIL is an integral/scaffold protein of the Cajal bodies and consequently mediates the assembly of small nuclear ribonucleoproteins (RNPs) and their related essential functions such as mRNA splicing (36, 37).

MOB3B and MOB3C shared four additional proteins, three just between themselves (MINK1, DOCK11, and RPP14) and one (DOCK7) with MOB1A/B. MINK1 is implicated in different signaling networks such as mTORC2 and STRIPAK and was suggested as another LATS kinase (28, 38, 39). DOCK7/11 are guanine nucleotide exchange factors that control RAC1 and CDC42 to regulate cytoskeletal dynamics (40). Importantly, the connection between DOCK7 to MOB1A/B was described before (16). RPP14 is a protein subunit of the RNase P complex (see later). Taken together, these data demonstrate uniqueness of the interactome of the MOB3 subfamily despite the high sequence similarity between them (82% between MOB3A and MOB3B; 74% between MOB3A and MOB3C; and 72% between MOB3B and MOB3C).

### BioID reveals novel proximity interactions of MOB proteins

**Figure 4:**
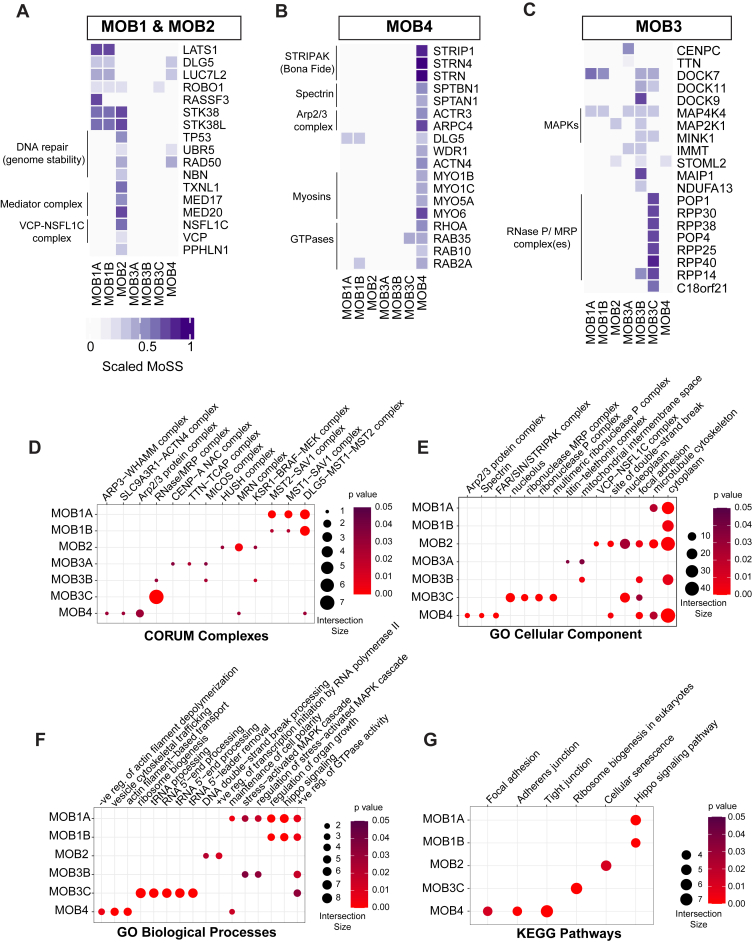
**BioID reveals novel interactors of the MOB proteins.***A*–*C*, heatmaps highlighting proteins in the proximity of MOB1A/B, MOB2 (*A*), MOB4 (*B*), and MOB3A/B/C (*C*). *D*–*G*, dot plots displaying selected terms from over-represented CORUM complexes (*D*), Gene Ontology (GO) cellular components (*E*), GO biological processes (*F*), and KEGG pathways (*G*). BioID, biotinylation identification; KEGG, Kyoto Encyclopedia of Genes and Genomes; MOB, monopolar spindle-one-binder.

Toward defining candidate novel interactors of the MOB proteins and exploring uncharacterized members, we undertook two approaches: (i) defining preys that were not recalled from the BioGrid database (Table S2) and (ii) collective functional analysis of the proximity interactome of each MOB to explore whether there is an enrichment for specific protein complexes, cellular components, signaling pathways, and/or a specific biological process (Table S3). Expectedly, given the high number of previously defined interactors of MOB1A/B (Fig. 2*B*), we did not define novel potential interactors that passed the stringent selection thresholds with respect to spectral counts and statistical significance (Fig. 4*A*).

MOB2 has recently been connected to DNA damage response and repair based on its interaction with RAD50 (41, 42), which belongs to the MRE11A–RAD50–NBN complex (43). This notion is reinforced in our dataset and extended further by capturing RAD50 and NBN in addition to TP53 and UBR5 in the vicinity of MOB2 (Fig. 4, *A* and *D*). Also, as part of the DNA damage response, we identified TXNL1, which regulates levels of the DNA repair protein XRCC1 (44, 45, 46), as a MOB2-specific prey (Fig. 4*A*). The collective functional analysis of MOB2 interactome recalled components of the HUSH and valosin-containing protein (VCP)–NSFL1C complexes (Fig. 4, *D* and *E*). Periphilin 1 (PPHLN1) forms the HUSH complex along with TASOR and MPP8 to mediate epigenetic silencing (47, 48). VCP and its cofactor NSFL1C are required for membrane fusion and consequently the Golgi reassembly/regeneration (49, 50, 51). We identified PPHLN1, VCP, and NSFL1C as MOB2-specific prey proteins (Fig. 4*A*). These observations collectively confirm the diverse nature of the defined MOB2 proximity interactome.

MOB4 proximity interactome was enriched for protein complexes, cellular components, and functions closely tied to the cytoskeleton and its regulation (Fig. 4, *D*–*G*). For instance, we identified SPTAN1 and SPTBN1 (Fig. 4, *B* and *E*), two subunits of the heterodimeric spectrin protein that organize(s) the cytoskeleton among other functions (52, 53). We also defined members of the actin-based motor myosin protein superfamily (MYO1B/C, MYO5A, and MYO6) (Fig. 4*B*) that was further reflected in highlighting actin-based transport and trafficking as over-represented biological processes in MOB4 interactome (Fig. 4*F*). While these highlighted preys were not recalled on BioGrid as MOB4 interactors (Table S2), some of them demonstrated a specificity pattern toward MOB4 similarly to its *bona fide* interactor(s), STRIP1 and STRNs (Fig. 4*B*).

Functional analyses did not pinpoint noteworthy enrichments in the MOB3A-specific interactome (Table S3). In contrast, MOB3B interactome recalled the mitogen-activated protein kinase signaling pathway as evident by capturing MAP4K4 (Fig. 4, *C* and *F*). Remarkably, a strong association between MOB3C and the RNase P complex or the closely related mitochondrial RNase P (MRP) complex was suggested in all the performed analyses. The RNase P and MRP are RNP complexes, each of which is composed of a catalytic RNA and ten protein subunits (eight of them are shared between the two complexes) to process pre-tRNAs and pre-rRNAs, respectively (54, 55). We found that seven of the shared protein subunits between these complexes (POP1, POP4, RPP14, RPP25, RPP30, RPP38, and RPP40) are exclusively among the top candidates in proximity to MOB3C (Fig. 4*C*). Moreover, the complex(es) and its related functions such as ribosomal biogenesis and tRNA processing were recalled with high representation and statistical significance in all the functional analyses (Fig. 4, *D*–*G* and Table S3). Independent analyses of the two screened cell lines suggested that these candidates are the top proximity hits for MOB3C (Fig. S1, *A* and *B*).

In summary, our BioID data robustly revealed the diversity of the MOB proteins in terms of spatial organization and functions of their potential interactors. As a proof of principle for the described proximity network, we chose to further validate the MOB3C–RNase P interaction experimentally given that MOB3C is currently of unknown function and that no previous connection between MOB proteins and the RNase P–MRP has been reported.

### MOB3C physically interacts with catalytically active RNase P complex

**Figure 5:**
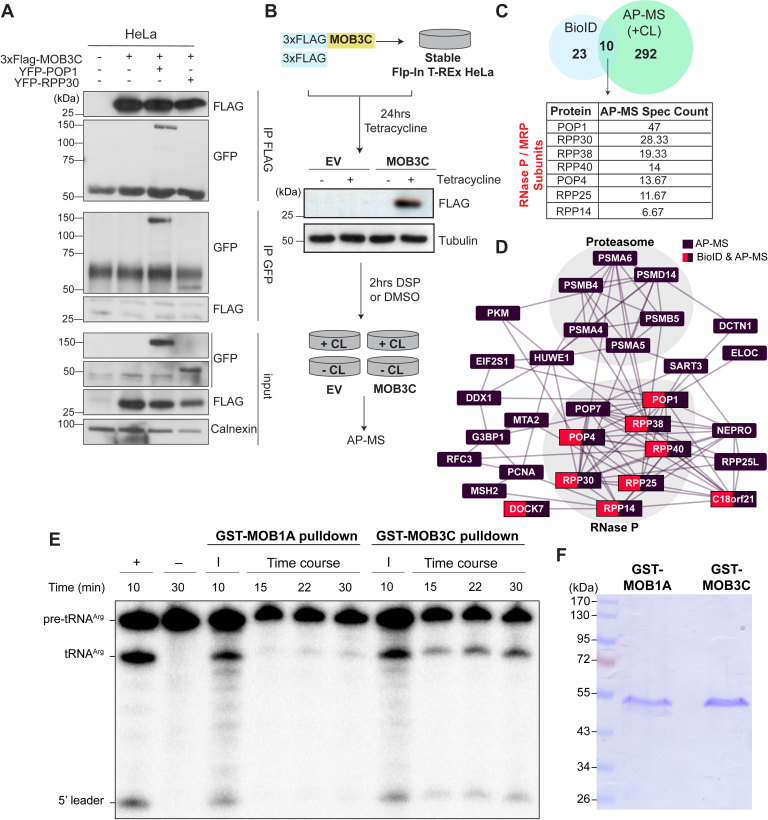
**MOB3C interacts with catalytically active RNase P complex.***A*, coimmunoprecipitation assay for YFP-tagged POP1 and RPP30 with FLAG-tagged MOB3C in HeLa cells. *B*, schematic outline for the affinity purification–mass spectrometry (AP–MS) with DSP crosslinking (CL). A Western blot analysis validating the used cell lines in AP–MS is shown. *C*, Venn diagram showing the commonly defined proteins in the BioID and crosslinked AP–MS datasets. The table displays the average spectral counts of each RNase P subunit defined in the AP–MS dataset. *D*, a proximity network of the RNase P protein subunits defined in both the MOB3C BioID and AP–MS datasets in reference to BioGrid (see the Experimental procedures section). *E*, pre-tRNA cleavage assay of the pull-down experiments using GST-MOB1A or GST-MOB3C. Time-course reactions showing RNase P activity when GST-MOB3C, but not GST-MOB1A, was used. +: a positive control of pre-tRNA^Arg^ cleaved by recombinant *Escheichia coli* RNase P; –: a negative control of pre-tRNA^Arg^ without enzyme; I: input. *F*, SDS-PAGE analysis confirms the presence of GST-MOB3C and GST-MOB1A post-pulldown. DSP, dithiobis (succinimidyl propionate); EV, empty vector; GST, glutathione-*S*-transferase; MOB, monopolar spindle-one-binder.

We opted to validate the predicted interaction between MOB3C and the protein subunits of RNase P by performing coimmunoprecipitation (IP) experiments with individual subunits of the RNase P. Upon immunoprecipitating 3xFLAG-MOB3C, we were able to detect YFP-POP1 but not YFP-RPP30 (Fig. 5*A*), the two subunits with the highest abundance in the MOB3C interactome (Fig. S1, *A* and *B*). Furthermore, we failed to recover MOB3C in POP1 or RPP30 immunoprecipitates (Fig. 5*A*). To account for the possibility that MOB3C interacts with the assembled holoenzyme (*i.e.*, the complete RNP complex), we undertook an affinity purification–mass spectrometry (AP–MS) approach that included a chemical crosslinking step to validate the interaction. We generated tetracycline-inducible HeLa Flp-In T-REx cells expressing either 3xFLAG-MOB3C or 3xFLAG (Fig. 5*B*) and induced the expression of MOB3C for 24 h followed by a 2 h treatment of dithiobis(succinimidyl propionate) (DSP) to crosslink the interactors before IP and MS analysis (Fig. 5*B*). The different replicates from all the conditions showed robust tightness as suggested by high Spearman correlation score (Fig. S2), giving confidence in the generated dataset. Toward our central question of whether MOB3C interacts with the RNase P protein subunits, we compared the BioID and AP–MS (with crosslinking) datasets and found only ten shared proteins. Among those ten were the seven RNase P–MRP protein subunits, which notably were not retrieved in the AP–MS condition without crosslinking (Fig. 5*C* and Table S4). Furthermore, the RNase P complex and its tRNA processing function were identified among the top represented CORUM complexes and Gene Ontology Biological Processes in the crosslinked AP–MS dataset, respectively (Fig. S3, *A* and *B* and Table S5). Overall, these results confirmed that MOB3C interacts with the RNase P RNP complex.

The RNase P holoenzyme and its subunits might interact with different proteins or complexes to mediate different functions beyond the canonical tRNA processing role (56, 57). This notion is only starting to be unraveled by building the RNase P proximity network. Therefore, we asked whether the MOB3C BioID and crosslinked AP–MS datasets can intersect with such a network. Interestingly, our assembled network recalled previously identified interactors of the RNase P complex, or at least some of its subunits, such as NEPRO (58), PCNA, RFC, MSH2, and different subunits of the proteasome (56, 59) (Fig. 5*D*). Notably, within this network was C18orf21, which was identified as one of the ten common prey proteins between the MOB3C AP–MS and BioID.

To assess whether MOB3C interacts with a functional RNase P complex, we pulled down RNase P using glutathione-*S*-transferase (GST)-MOB3C or GST-MOB1A (negative control) as baits and assayed for precursor tRNA cleavage activity. We observed that significant pre-tRNA^Arg^ (n-Tr21) cleavage activity was pulled down by MOB3C compared with the MOB1A negative control (Fig. 5*E*). We also confirmed that both GST-MOB3C and GST-MOB1A were pulled down with similar efficiency by glutathione beads (Fig. 5*F*). Collectively, these results strongly suggest that MOB3C interacts with a catalytically active RNase P holoenzyme.

## Discussion

The MOB proteins, at least the well-studied family members, mediate their functions through engaging in protein–protein interactions and assembling complexes. Here, we systematically uncovered the proximity network of the MOB family and found a minimal overlap between different subfamilies (*e.g.*, MOB1 *versus* MOB3). Importantly, the interactomes of the three MOB3 proteins are different, arguing against considering them as a subfamily with a unified function (8, 15). MOB3A and MOB3C are upregulated in gliomas, and combined depletion of all three human MOB3 proteins halted the proliferation of a glioblastoma cell line *in vitro* and *in vivo* (15). The study concluded that the human MOB3s mediate a protumorigenic effect. However, the authors did not assess the effect of individual depletions of MOB3A/B/C, but instead depleted simultaneously all three MOB3s, assuming they offer functional redundancy. Our unbiased proteomic screens argue against such a notion.

In addition to comparing the subfamilies to each other, our screens identified novel interactors for different MOBs that potentially guide the search for their molecular functions. Importantly, BioID showed superiority in revealing such interactions when compared with traditional methods, such as the yeast two-hybrid assays used to reveal MOB2 interactors or AP–MS strategy used with human MOBs (17, 41). Some of the identified interactions extend previous findings (*e.g.*, connecting MOB2 to DNA repair proteins and MOB4 to cytoskeletal remodelers), whereas others spawn new directions (*e.g.*, connecting different MOBs to mitogen-activated protein kinases that might potentially converge on the Hippo pathway). The latter feeds into the unresolved relationship between MOB3 proteins and this pathway. In agreement with our BioID screens, previous co-IP assays or AP–MS screens suggested that MOB3 proteins do not interact with or activate LATS or STK38 (17, 27). Meanwhile, an interaction between MOB3 proteins and the Hippo pathway core kinase, MST1, was reported only under specific experimental conditions (8, 15), which our screens did not recall. Here, we report the existence of the noncanonical Hippo regulators MAP4K4/6 and PTPN14 in the vicinity of MOB3 proteins. However, such notions would require thorough experimental validations in future studies, since proteins defined in BioID screens are not necessarily direct interactors with the bait protein.

The biological function of MOB3C remains elusive. Recently, it was suggested to facilitate bypassing oncogene-induced senescence (8). However, these observations were obtained by studying a myristoylated form of MOB3C. Although myristoylation is a useful approach for revealing signaling circuits, as was the case for MOB1 (60, 61), it is limited in informing on the native MOB3C protein within physiological contexts. Here, through unbiased proteomics and subsequent GST-pulldowns, we reveal an unexpected connection between MOB3C and RNase P–MRP, two evolutionarily conserved and essential RNP complex(es). This observation rationalizes multiple questions. First, does the MOB3C–RNase P interaction require other intermediary proteins? Second, because the seven protein subunits identified in the vicinity of MOB3C are shared between the RNase P and RNase MRP (62), does MOB3C interact preferentially with one or both RNPs? Either way, the reason and consequences behind this interaction should be addressed by future studies. One possibility is MOB3C regulating the complex(es)’ catalytic activity or substrate specificity. This would go in line with the view that the H1 RNA of the RNase P accumulated additional protein subunits through evolution to tune its affinity toward different substrates (24, 63, 64). Alternatively, MOB3C might act as an adaptor to help assemble the holoenzyme and hence raises the question of which subunit(s) it binds. In this direction, it is worth noting the failure of our BioID and AP–MS to retrieve three subunits of human RNase P (RPP20, RPP21, and POP5) in the vicinity of MOB3C. This finding is unexpected given the long-range diffusibility of biotin and the proximity of RPP20, RPP21, and POP5 to their respective partners in the fully assembled complex (RPP25 from the RPP20–RPP25 heterodimer; RPP29 and RPP38 from the RPP29–RPP21–RPP38 heterotrimer; RPP14 and RPP30 from the RPP14–(RPP30)_2_–POP5 heterotetramer) (62). We consider two reasons for not retrieving RPP20, RPP21, and POP5 in BioID and AP–MS, both of which depend on solvent-exposed Lys residues. First, the cryo-EM structure of human RNase P (62), albeit not modeled for all the Lys residues, reveals very few solvent-exposed Lys residues in RPP20, RPP21, and POP5; notably, these three RNase P proteins also have the lowest number and percent composition of Lys residues among all ten RNase P subunits, implying a lower baseline likelihood for modification/crosslinking. Second, it is possible that a low ionization efficiency of peptides derived from these proteins resulted in false negatives during MS.

The specificity of the RNase P complex for MOB3C was clear in our BioID interactomes, a convincing demonstration that individual MOB3 proteins have evolved distinct functions. While the MOB3 proteins share 72 to 82% identity at the amino acid level and the AlphaFold predicted structures are indistinguishable (average backbone rmsd of 0.25 Å), MOB3C has acquired several additional charged residues, particularly arginine, across a broad region of the protein that includes an unstructured loop that could complex with an RNP such as RNase P (Fig. S4). The 12 arginine residues on this face of MOB3C are significantly more than those found in MOB3A (6) or MOB3B (4), a potential rationalization for their differential binding to the RNase P complex.

In summary, our study not only provides a resource for exploring new interactors and consequently functions of the seven human MOB proteins but also puts forward an interesting connection between the MOB family and RNA processing.

## Experimental procedures

### Cell lines and culture

HEK293 Flp-In T-REx cells were purchased from ThermoFisher Scientific, whereas HeLa Flp-In T-REx cells were a gift from S. Taylor (University of Manchester). All cell lines were cultured in Dulbecco’s modified Eagle’s medium (Wisent; catalog no.: 319-005-CL) supplemented with 10% fetal bovine serum and 1% penicillin–streptomycin antibiotics (Wisent).

### Generating stable cell lines and BioID pipeline and analysis

To generate gateway-compatible sequences, the seven human MOB complementary DNA sequences (including the stop codons) flanked by the attB sequences were synthesized (Twist Bioscience). These complementary DNAs (cDNAs) were consequently recombined into the pDONR-221 vector and then shuttled into the pcDNA5-FRT backbone vector expressing an abortive mutant of BirA (BirA∗) tagged with FLAG using the gateway recombination cloning system (16, 65). Generating the stable Flp-In T-REx expressing BirA∗-FLAG-MOBs or BirA∗-FLAG or BirA∗-FLAG-EGFP was done according to the manufacturer protocol and as described here (65). For validating the construction of the cell lines, cells were seeded into 6-well plates (Corning) and incubated overnight. The following day, cells were induced with tetracycline (1 μg/ml) and/or treated with biotin (50 μM) for 24 h. Cells were then lysed with ice-cold radioimmunoprecipitation assay buffer (1% Nonidet P-40, 0.5% deoxycholic acid, 0.1% SDS, 150 mM NaCl, 5 mM EDTA, 50 mM Tris [pH 7.5]) supplied with NaF (5 mM), Na3VO_4_ (1 mM), and 1× complete protease inhibitor (Roche). Western blotting was done using the following concentrations of the antibodies: monoclonal anti-FLAG M2-horseradish peroxidase (HRP) (1:10,000 dilution; Sigma, catalog no.: A8592) and streptavidin–HRP (1:25,000 dilution; BD Biosciences, catalog no.: 554066) in Tris-buffered saline (TBS) solution (1% bovine serum albumin and 0.1% Tween-20).

For BioID, cells were seeded in 15 cm plates (Sarstedt) to reach 70 to 80% confluence. Subsequently, simultaneous induction with tetracycline (1 μg/ml) and treatment with biotin (50 μM) was done 24 h prior to processing the cells as we previously detailed (20, 65) with no modifications.

MS data were analyzed as we previously described (20). Briefly, the raw data were analyzed with ProHits (66). The MOBs’ two datasets (HEK293 and HeLa) were compared with their respective controls (BirA-FLAG and EGFP-BirA-FLAG). To define interaction statistics, we used SAINTexpress (version 3.6.1) (67) through ProHits (66) by using the following parameters: iProphet protein probability ≥0.9 and unique peptides ≥1. Each of the proteomics datasets (HEK293 and HeLa) was compared individually *versus* their negative controls (EGFP and empty vector). SAINT analyses were performed using the following settings: nControl:3 and nCompressBaits:3 (no baits compression). Preys’ bait specificities (WD-score) were calculated for each cell line separately by using the CompPass algorithm (68) after we uploaded the SAINT results to the ProHHits Prey Specificity online tool (https://prohits-viz.lunenfeld.ca/Specificity/). Interactions having a Bayesian false discovery rate (BFDR) ≤0.01 were considered as statistically significant. Next, we estimated the MoSS index for each prey by summing both their cell lines’ WD-scores. The data were log2-transformed and rescaled on a range of 0.1 to 1.0 in R (www.r-project.org) with the Scales package. After we assigned a value of zero to unidentified interactions, we created a heatmap with the pheatmap package in R. An UpSet plot was created with the UpSetR package in R by enumerating nonredundant interactions identified by BioID from both HEK293 and HeLa cell lines. Enumerations of interactions were calculated on statistically significant preys (BFDR ≤0.01) identified in both cell lines for all seven MOB baits. We also enumerated interaction recalls by identifying within our statistically significant preys known MOB interactions from the human BioGrid database (version 4.4.208). A histogram of these enumerations was created in R by using the ggplot2 package. The R package gprofiler2 was used to analyze the over-represented Gene Ontology terms, Kyoto Encyclopedia of Genes and Genomes pathways, and CORUM complexes (69) by statistically correcting *p* values with the FDR correction method. We selected statistically significant terms having adjusted *p* values <0.05 and presented the results in dotplots using the ggplot2 R package. The BioID and AP–MS results were imported in Cytoscape (version 3.9.1) (cytoscape.org) to build graphical representations of PPI networks. We then performed a network augmentation by extracting prey–prey interactions from the human BioGRID database (version 4.2.192) (70) and from Cytoscape’s PSICQUIC Web Service client (May 2021 release) through the IntAct and UniProt databases.

#### Dot plot analyses

Dot plots were produced through the ProHits-Viz online tool (https://prohits-viz.org/) with the generated SAINT output files, using a BFDR ≤0.01 as a statistical cutoff.

### AP–MS

MOB3C cDNA was shuttled into the pDEST 5′ Triple FLAG pcDNA5-FRT to generate an N-terminally FLAG-tagged MOB3C. Both this vector and the original pDEST5′ Triple FLAG pcDNA5-FRT vector were independently used to generate stable Flp-In T-REx HeLa cells expressing either FLAG-MOB3C or FLAG (empty vector), respectively (65). These two cell lines were seeded in 6-well plates overnight and then induced with tetracycline (1 μg/ml) for 24 h and then processed as described earlier to validate the expression of MOB3C.

For the AP–MS experiments, the generated Flp-In T-REx HeLa cells were seeded in 15 cm plates overnight and induced with tetracycline (1 μg/ml) for 24 h before processing for the AP–MS. For the crosslinking, DSP (ThermoFisher Scientific; catalog no.: 22585) was dissolved in dimethyl sulfoxide for a 100 mM stock solution, and a warm 1% DSP solution in calcium- and magnesium-free Dulbecco's PBS (ThermoFisher Scientific; catalog no.: 21600010) was added on the cells for 2 h on an ice-water bath for crosslinking (71). Subsequently, cells were incubated for 15 min on ice with a DSP quenching solution (20 mM Tris [pH 7.5] in Dulbecco's PBS). The AP–MS experiments were done as previously reported (16) with the following modifications. Briefly, cells were collected and lysed in 0.1% NP-40 lysis buffer (100 mM KCl, 50 mM Hepes–KOH [pH 8.0], 2 mM EDTA, and 10% glycerol) supplemented with 1× protease inhibitor cocktail, 1 mM DTT, and 1 mM PMSF. AP was carried out using 20 μl of anti-FLAG M2 magnetic beads (Sigma; catalog no.: M8823-1ML) per condition. The on-bead proteins were first diluted in 2 M urea/50 mM ammonium bicarbonate, and on-bead trypsin digestion was performed overnight at 37 °C. The samples were then reduced with 13 mM DTT at 37 °C, cooled for 10 min, and alkylated with 23 mM iodoacetamide at room temperature for 20 min in the dark. Trifluoroacetic acid was used to acidify supernatants. MCX cartridges (Waters Oasis MCX 96-well Elution Plate) were then used to clean the supernatants from residual detergents and reagents according to the manufacturer’s instructions. After elution in 10% ammonium hydroxide/90% methanol (v/v), samples were dried in a Speed-vac, reconstituted under agitation for 15 min in 22 μl of 2% acetonitrile–1% formic acid (FA), and loaded onto a 75 μm i.d. × 150 mm Self-Pack C18 column installed in the Easy-nLC II system (Proxeon Biosystems). The solvents used for chromatography were 0.2% FA in water (solvent A) and 0.2% FA in acetonitrile (solvent B). Peptides were eluted with a two-slope gradient at a flow rate of 250 nl/min. Solvent B first increased from 2 to 34% in 80 min and then from 34 to 80% B in 12 min. The HPLC system was coupled to an Orbitrap Fusion mass spectrometer (Thermo Scientific) through a Nanospray Flex Ion Source. Nanospray voltage was set to 1.3 to 1.7 kV, meanwhile the S-lens voltage was set to 60 V. Capillary temperature of 225 °C was set. Full scan MS survey spectra (*m/z* 360–1560) in profile mode were acquired in the Orbitrap with a 120,000 resolution and a 3e5 target value. The 25 most intense peptide ions were fragmented in the higher energy collision-induced dissociation cell and analyzed in the linear ion trap with a target value at 1e4 and a normalized collision energy at 29. Target ions selected for MS–MS fragmentation were dynamically excluded for 20 s after two MS2 events.

### Immunostaining and confocal microscopy

HeLa Flp-In T-REx cells were plated on coverslips, and simultaneous induction with tetracycline (1 μg/ml) and treatment with biotin (50 μM) were done 24 h prior to cell fixation with 3.7% formaldehyde in CSK buffer (100 mM NaCl, 300 mM sucrose, 3 mM MgCl_2_, 10 mM Pipes, pH 6.8). Then, cells were permeabilized with 0.2% Triton X-100 in PBS for 5 min before incubation with rabbit polyclonal FLAG antibody (Cell Signaling Technology; catalog no.: 2368) at 1:1000 dilution in the wash buffer (1%  bovine serum albumin and 0.1% Triton X-100 in TBS), rinsed five times with wash buffer, and incubated for 30 min with Alexa Fluor 488–conjugated chicken anti-rabbit (Life Technologies; catalog no.: A-21441) at 1:500 dilution together with Hoechst 33342 (Invitrogen; catalog no.: H3570) at 1:10,000 dilution and Alexa Fluor 633-conjugated streptavidin (Thermo Fisher Scientific; catalog no.: S21375) at 1:500 dilution in wash buffer. Cells were washed ten times with the wash buffer and one time with water. Coverslips were mounted with Mowiol. Images were acquired with a Carl Zeiss LSM700 laser scanning confocal microscope (Carl Zeiss MicroImaging) equipped with a plan-apochromat 63×/1.4 numerical aperture objective and operated with ZenBlack 2009.

### Co-IP experiments

YFP-RPP30-C1 was a gift from Susan Janicki (Addgene plasmid #134547). YFP-POP1 expression vector was generated by amplifying the POP1 cDNA from pCMVh-POP1-3xFLAG (gift from Martin Dorf; Addgene #53968) with flanking XhoI and SalI restriction sites and ligating it into the multicloning site of YFP-C1 backbone vector by swabbing out the YFP-RPP30. Primers used are XhoI-POP1 (forward: taagcaCTCGAGaaatgtcaaatgcaaaagaaag) and POP1-SalI (reverse: tgcttagtcgactcacacctcaatagcaatcctcg). Flp-In T-REx HeLa–expressing FLAG-MOB3C were transfected with 10 μg of either YFP-RPP30 or YFP-POP1 using Lipofectamine 2000 (Invitrogen) according to the manufacturer’s instructions. About 24 h after transfection, the expression of MOB3C was induced with tetracycline (1 μg/ml) for 24 h. About 48 h after transfection and 24 h after tetracycline induction, cells were lysed with CHAPS lysis buffer (0.5% CHAPS, 1 mM MgCl_2_, 1 mM EGTA, 10 mM Tris [pH 7.5], 10% glycerol, 5 mM β-mercaptoethanol, and 5 mM NaF) supplemented with 1× complete protease inhibitor and 1 mM Na_3_VO_4_ as previously reported (72). For IP, 20 μl/condition of either anti-FLAG M2 affinity gel (Sigma) or Protein G agarose beads (Genscript) were washed three times with 1 ml of IP buffer (72) (0.1% Triton X, 20 mM Tris [pH 7.5], 10 mM MgCl_2_, 150 mM KCl, and 10% glycerol). Beads were then incubated for 2 h at 4 °C with 1 mg of protein lysate alone or with the addition of 1 μl of GFP antibody (Life Technologies) for the FLAG and GFP IP, respectively. Immunoprecipitates were then washed three times with the IP buffer and denatured in 6× sample buffer (350 mM Tris–HCl [Ph 6.8], 10% SDS, 30% glycerol, 0.1% bromophenol blue, and 10% β-mercaptoethanol). Lysates and immunoprecipitates were then processed for immunoblotting. 1× Wet-transfer buffer (from 10× stock buffer; 25 mM Tris, 192 mM glycine in water) and nitrocellulose membranes (Bio-Rad; catalog no.: 1620115) were used for the transfer step. The following concentrations: FLAG M2-HRP at 1:10,000 dilution (Sigma; catalog no.: A8592), GFP at 1:2000 dilution (Life Technologies; catalog no.: A-11122), and Calnexin E10 at 1:500 dilution (Santa Cruz; catalog no.: sc-46669) in TBS solution were used for probing the membranes.

### Purification of GST-MOB3C and GST-MOB1A

The plasmid pTH35-GST-MOB3C was used to transform *Escherichia coli* BL21 RIL Codon Plus (Agilent) cells. A single colony was used to inoculate 5 ml LB medium containing 100 μg/ml carbenicillin, 25 μg/ml chloramphenicol, and 1% (w/v) glucose, and the culture was grown overnight at 37 °C with shaking. This seed culture was used to inoculate 500 ml LB medium (supplemented with carbenicillin and chloramphenicol as aforementioned). The culture was incubated at 37 °C with shaking until an absorbance of ∼0.6 at 600 nm before induction with 0.4 mM IPTG and growth continued at 15 °C overnight. The cells were harvested by centrifugation (6000*g*, 15 min, 4 °C), and the pellet was resuspended in 20 ml of buffer A (1× PBS [pH 7], 0.5 M NaCl, 1 mM PMSF, and 1 mM DTT). The resuspended cells were lysed by sonication, and the cell lysate was cleared by centrifugation (23,000*g*, 30 min, 4 °C). The resulting supernatant was added to 800 μl of glutathione agarose (Thermo Scientific), which was equilibrated in lysis buffer. After nutating at 4 °C for 1 h, the resin was pelleted at 700*g* for 2 min and washed extensively with wash buffer (1× PBS [pH 7], 0.5 M NaCl, and 1 mM DTT), before eluting twice with 3 ml and then 2 ml of elution buffer (50 mM Tris–HCl [pH 8], 150 mM NaCl, 1 mM DTT, and 20 mM reduced glutathione). After confirming the presence of GST-MOB3C by SDS-PAGE, the eluents were pooled and diluted with buffer A0 (50 mM Tris–HCl [pH 7.5], 1 mM DTT) to reduce the NaCl in the sample to 25 mM. The sample was then loaded on a 1 ml Heparin HP Sepharose column (GE Healthcare), which was equilibrated with buffer A50 (50 mM Tris–HCl [pH 7.5], 50 mM NaCl, and 1 mM DTT). The column was washed extensively with buffer A50, and the protein was eluted with a linear 0 to 1 M NaCl gradient using buffer A1000 (50 mM Tris–HCl [pH 7.5], 2M NaCl, and 1 mM DTT) and an AKTA FPLC purifier (GE Healthcare). After SDS-PAGE analysis, fractions containing GST-MOB3C were pooled and dialyzed overnight at 4 °C against 1 l of buffer C (20 mM Tris–HCl [pH 8], 150 mM NaCl, 14.3 mM β-mercaptoethanol, and 10% glycerol). The dialyzed sample was concentrated using a 5 kDa Amicon centrifugal filter (Millipore) with molecular weight cutoff of 5 kDa, and the final protein concentration was determined by measuring Abs280 using a Nanodrop spectrophotometer and using an extinction coefficient of 75,750 M^−1^cm^−1^ (ExPASy ProtParam Tool) (73). The protein was flash frozen and stored at −80 °C until use. A similar protocol was followed to obtain GST-MOB1A using plasmid pTH35-GST-MOB1A. The concentration of GST-MOB1A was determined using an extinction coefficient of 72,770 M^−1^ cm^−1^ (ExPASy ProtParam Tool) (73).

### Pulldown of HeLa RNase P using GST-MOB3C and GST-MOB1A

HeLa cell pellets (∼10^7^ cells/pellet) were harvested from confluent 15 cm plates. First, plates were washed with PBS, and then cells were scraped in PBS and pelleted by centrifugation. Pellets were washed three times in PBS and stored at −80 °C until use. Cell pellets were gently resuspended in 750 μl of lysis buffer (20 mM Tris–HCl [pH 8], 100 mM NaCl, 5 mM MgCl_2_, 1% glycerol, 14.3 mM β-mercaptoethanol, and 0.2 mM PMSF). One-third of the resuspended cells was lysed by sonication, and the remaining two-thirds were stored at −80 °C for future use. The cell lysate was cleared by centrifugation (20,000*g*, 20 min, 4 °C). One-half of the supernatant was mixed with ∼120 pmol GST-MOB3C and the other half with ∼120 pmol GST-MOB1A. Both samples were incubated at 37 °C (with nutation) for 15 min and then on ice for 30 min. Two microliters of each sample were saved as the input (I). Each sample was then added to 80 μl glutathione agarose beads (Thermo Scientific), which had been pre-equilibrated in lysis buffer. All samples were nutated at 4 °C for 90 min before the beads were harvested and washed twice with 500 μl of wash buffer (20 mM Tris–HCl [pH 8], 100 mM NaCl, 5 mM MgCl_2_, 14.3 mM β-mercaptoethanol, and 0.005% [v/v] NP-40). One-third of the beads was used for RNase P activity assay with precursor n-Tr21 (pre-tRNA^Arg^) as the substrate (74). The beads were mixed with 10 μl reaction buffer (20 mM Tris–HCl [pH 8], 14.3 mM β-mercaptoethanol, 100 mM KCl, 1 mM MgCl_2_, 20 nM pre–n-Tr21, a trace amount of which was internally labeled with [α-^32^P]-GTP). The reaction was nutated at 37 °C, and 3 μl aliquots were withdrawn at 15, 22, and 30 min and quenched with 10 μl loading dye (7 M urea, 20% [v/v] phenol, 0.2% [w/v] SDS, 5 mM EDTA, 0.05% [w/v] bromophenol blue, and 0.05% [w/v] xylene cyanol). The reaction products were then separated on a denaturing PAGE gel (10% [w/v] polyacrylamide and 7 M urea) and visualized using a Typhoon Phosphorimager (GE Healthcare). One-fifth of the beads was analyzed by SDS-PAGE and Coomassie blue staining to confirm the presence of GST-MOB1A and GST-MOB3C in the precipitates. The pulldown experiment was independently repeated two times, and the same trend was observed. Only one representative gel is shown.

## Data availability

The raw proteomics data of the BioID and AP–MS screens that are presented in this article have been deposited into the MassIVE repository (https://doi.org/10.25345/C5H98ZQ09). As stated in the Supporting information section, all the data and analyses linked to these datasets are presented in Tables S1–S5.

## Supporting information

This article contains supporting information.

## Conflict of interest

The authors declare that they have no conflicts of interest with the contents of this article.

## Supporting information

Supporting Tables S4 and S5

Supporting Figure S1 **BioID reveals new interactors for MOB3C.***A* and *B*, dot plots showing the enrichment of the protein subunits of the RNase P/MRP complexes in the vicinity of MOB3C in HeLa (*A*) and HEK293 (*B*) cells.

Supporting Figure S2 **Quality control for the generated AP-MS datasets.** The biological replicates of the DSP-crosslinked MOB3C condition showed high Spearman correlations (0.91) with each other confirming tightness between these replicates. REP, replicate.

Supporting Figure S3 **Functional analyses for MOB3C interacting proteins.** The list of statistically significant interactors of MOB3C from the cross-linked AP-MS dataset was used to define the top ten overrepresented CORUM complexes (by adj. *p*-values) (*A*), and Gene Ontology Biological Processes (BP) (*B*).

Supporting Figure S4 **Structural insights into the MOB3 subfamily.***A*, amino acid sequences of the 3 MOB3 proteins with the arginine residues highlighted in red. *B*, alpha Fold prediction of the three MOB3 proteins’ structure highlighting higher number of arginine residues in MOB3C in comparison to MOB3A/B. Highlighted arginine residues from panel (*A*) are enumerated in (*B*).
