# Supplementary figures and images for: Mapping the MOB proteins’ proximity network reveals a unique interaction between human MOB3C and the RNase P complex
Source: J Biol Chem. 2023 Aug 1;299(9):105123. doi: 10.1016/j.jbc.2023.105123 (PMC10480535; doi:10.1016/j.jbc.2023.105123)

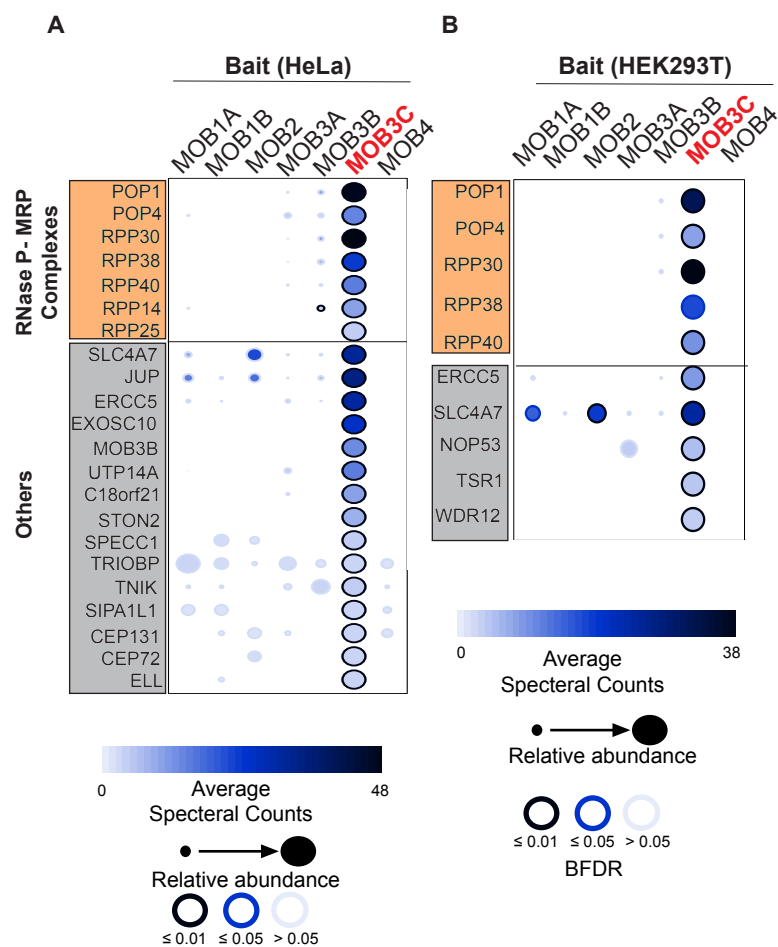

**Figure S1**

Supplement: Supporting Figure S1 — BioID reveals new interactors for MOB3C.A and B, dot plots showing the enrichment of the protein subunits of the RNase P/MRP complexes in the vicinity of MOB3C in HeLa (A) and HEK293 (B) cells. [file mmc3.pdf]

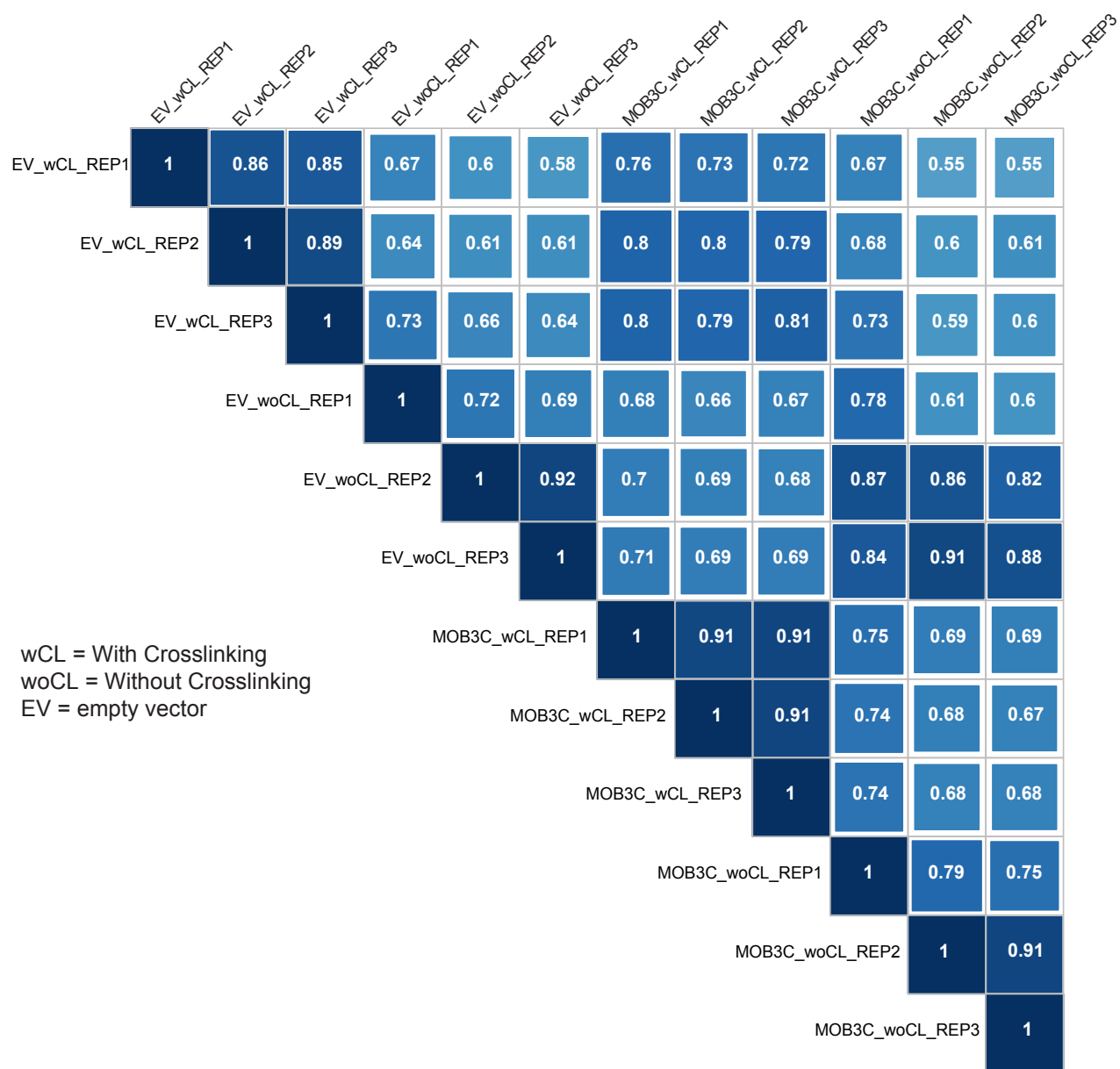

**Figure S2**

Supplement: Supporting Figure S2 — Quality control for the generated AP-MS datasets. The biological replicates of the DSP-crosslinked MOB3C condition showed high Spearman correlations (0.91) with each other confirming tightness between these replicates. REP, replicate. [file mmc4.pdf]

**A**

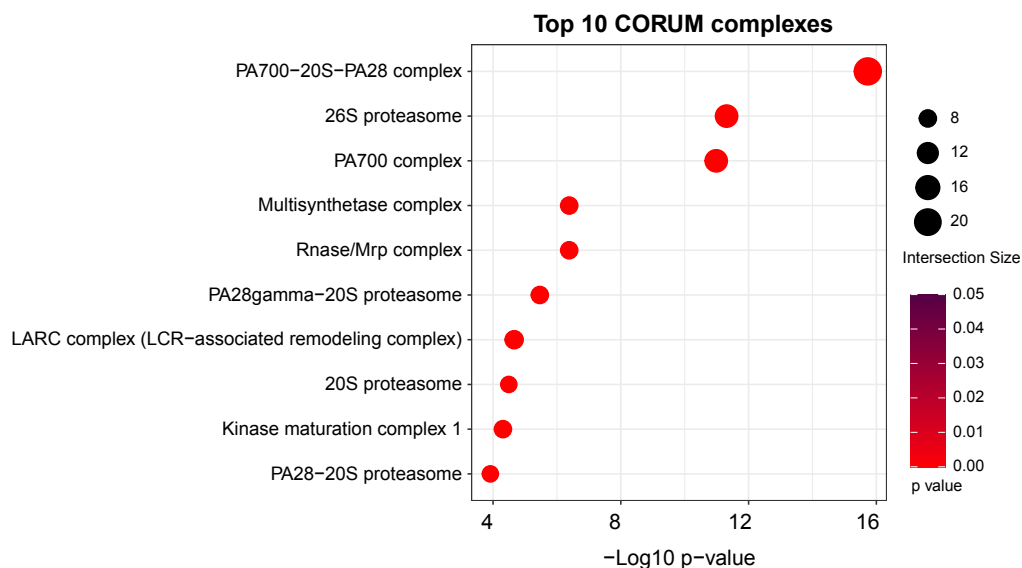

**B**

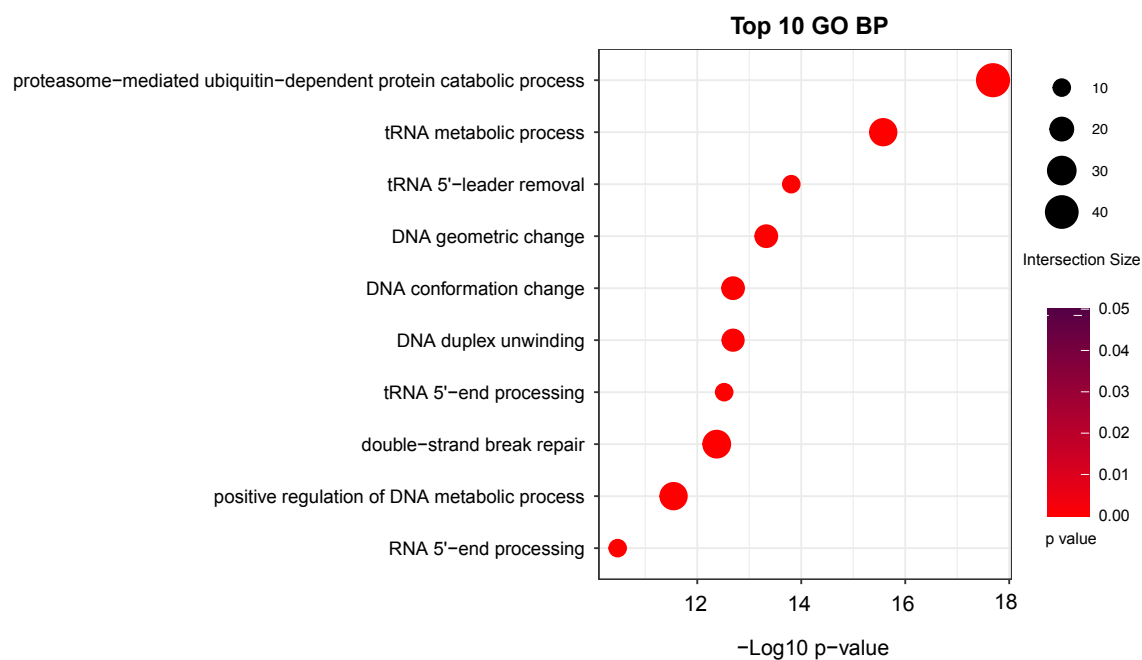

**Figure S3**

Supplement: Supporting Figure S3 — Functional analyses for MOB3C interacting proteins. The list of statistically significant interactors of MOB3C from the cross-linked AP-MS dataset was used to define the top ten overrepresented CORUM complexes (by adj. p-values) (A), and Gene Ontology Biological Processes (BP) (B). [file mmc5.pdf]
