# Supplementary material for: Mapping the MOB proteins’ proximity network reveals a unique interaction between human MOB3C and the RNase P complex
Source: J Biol Chem. 2023 Aug 1;299(9):105123. doi: 10.1016/j.jbc.2023.105123 (PMC10480535; doi:10.1016/j.jbc.2023.105123)
Supplement: Supporting Figure S4 — Structural insights into the MOB3 subfamily.A, amino acid sequences of the 3 MOB3 proteins with the arginine residues highlighted in red. B, alpha Fold prediction of the three MOB3 proteins’ structure highlighting higher number of arginine residues in MOB3C in comparison to MOB3A/B. Highlighted arginine residues from panel (A) are enumerated in (B). [file mmc6.pdf]

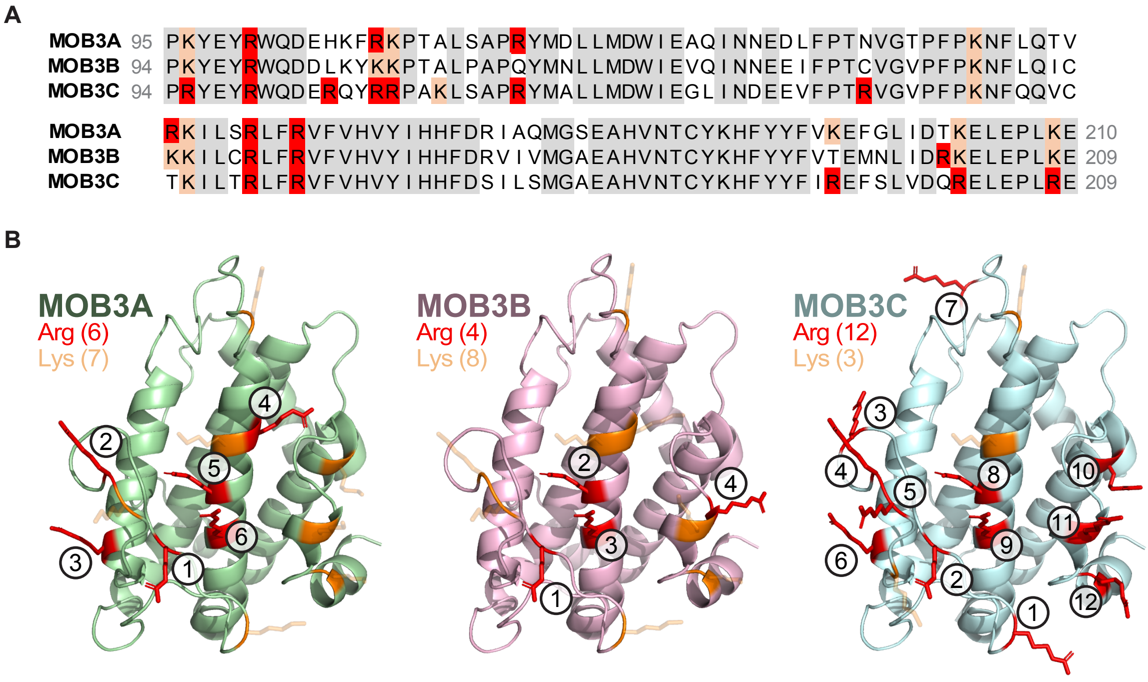
A
MOB3A 95 PKYEYRWQDEHKFRRKPTALSAPRYMDLLMDWIEAQINNEDLFPTNVGTPFPKNFLQTV
MOB3B 94 PKYEYRWQDDLKYKKPTALPAPQYMNLLMDWIEVQINNEEIFPTCVGVPFPKNFLQIC
MOB3C 94 PRYEYRWQDERQYRRPAKLSAPRYMALLMDWIEGLINDEEVFPTRVGVPFPKNFQQVC
MOB3A RKILSRLFRVFVHVYIHHFDRIAQMGSEAHVNTCYKHFYYFVKEFGLIDTKELEPLKE 210
MOB3B KKILCRLFRVFVHVYIHHFDRVIVMGAEAHVNTCYKHFYYFVTEMNLIDRKELEPLKE 209
MOB3C TKILTRLFRVFVHVYIHHFDSILSMGAEAHVNTCYKHFYYFIREFSLVDQRELEPLRE 209
B
MOB3A
Arg (6)
Lys (7)
MOB3B
Arg (4)
Lys (8)
MOB3C
Arg (12)
Lys (3)

Figure S4
